# Polyurethanes Made with Blends of Polycarbonates with Different Molecular Weights Showing Adequate Mechanical and Adhesion Properties and Fast Self-Healing at Room Temperature

**DOI:** 10.3390/ma17225532

**Published:** 2024-11-13

**Authors:** Yuliet Paez-Amieva, Noemí Mateo-Oliveras, José Miguel Martín-Martínez

**Affiliations:** Adhesion and Adhesives Laboratory, University of Alicante, 03080 Alicante, Spain; yuliet.paez@ua.es (Y.P.-A.); noemi.mateo@ua.es (N.M.-O.)

**Keywords:** polycarbonate polyol, soft segments, length of the soft segments, intrinsic self-healing at 20 °C, blends of polyols, mechanical properties, adhesion

## Abstract

Dynamic non-covalent interactions between polycarbonate soft segments have been proposed for explaining the intrinsic self-healing of polyurethanes synthesized with polycarbonate polyols (PUs) at 20 °C. However, these self-healing PUs showed insufficient mechanical properties, and their adhesion properties have not been explored yet. Different PUs with self-healing at 20 °C, acceptable mechanical properties, and high shear strengths (similar to the highest ones reported in the literature) were synthesized by using blends of polycarbonate polyols of molecular weights 1000 and 2000 Da (CD1000 + CD2000). Their structural, thermal, rheological, mechanical, and adhesion (single lap-shear tests) properties were assessed. PUs with higher CD1000 polyol contents exhibited shorter self-healing times and dominant viscous properties due to the higher amount of free carbonate groups, significant carbonate–carbonate interactions, and low micro-phase separation. As the CD2000 polyol content in the PUs increased, slower kinetics and longer self-healing times and higher mechanical and adhesion properties were obtained due to a dominant rheological elastic behavior, soft segments with higher crystallinities, and greater micro-phase separation. All PUs synthesized with CD1000 + CD2000 blends exhibited a mixed phase due to interactions between polycarbonate soft segments of different lengths which favored the self-healing and mobility of the polymer chains, resulting in increased mechanical properties.

## 1. Introduction

Adhesives are crucial in both industrial applications and everyday life [1,2,3]. Particularly, polyurethane (PU) adhesives are extensively used in electronics, car manufacturing, aerospace applications, outdoor goods, wood processing, and the footwear industry [4,5]. Due to the exposure to thermal, mechanical, and chemical agents, as well as outdoor exposure, during use, PU adhesives can undergo damage and cracking, which may cause poor performance [6,7]. Self-healing is an attractive property that can extend the lifetime of PUs, contributing to reducing waste and re-use and avoiding resource and energy consumption. Therefore, there is a current demand for self-healing PUs and PU adhesives [5,7].

The self-healing mechanisms of PUs involve extrinsic and intrinsic healing [8,9,10,11,12,13,14]. The extrinsic self-healing mechanism involves the application of an external trigger (radiation, chemicals, etc.) and is limited by the release of functional additives and structural changes [7,15]. In contrast, the intrinsic self-healing mechanism operates without the need for any external repair agent [8], and dynamic reversible covalent or non-covalent reversible bonds are involved. This intrinsic self-healing involves various reactions [16,17], different chemicals [18,19], and different bonds [20,21].

Polyurethanes (PUs) are obtained by reacting polyol with isocyanate and a chain extender, resulting in a segmented structure made up of soft segments (long chains of polyol) and hard segments (urethane/urea groups) [22]. Phase separation occurs due to the differences in polarity and the chemical nature of these segments. This phase-separated microstructure is especially beneficial in self-healing PUs, as the soft phase facilitates the mobility of polymer chains while the hard phase provides physical and structural stability [15,23].

Whereas several self-healing PUs have been synthesized, only a few self-healing PU adhesives have recently been developed. Wang et al. introduced dynamic oxime–carbamate bonds into a cross-linked PU network to produce self-healing hot melt adhesives with high ultimate bond strengths (5.69 ± 1.30 MPa) [5]. Zhou et al. obtained catechol-functionalized PU elastomers intended for medical wound closing made with polytetramethylene ether glycol soft segments of different molecular weights (650, 1000, and 2000 Da), which exhibited significant self-healing at room temperature [24,25]. The shear bond strengths (adhesion) of these catechol-functionalized PUs were high in joints made with metallic substrates due to interactions with metal ions and metal oxides [26]. On the other hand, Xu et al. developed a semi-interpenetrating ionogel made with a poly(vinylidene fluoride-co-hexafluoropropylene) non-crosslinked network and a polyurethane crosslinked network that showed self-healing at room temperature after 30 min and shear strength values above 100 kPa [27]. Furthermore, Zhang et al. developed PUs formulated with hydroxyl-terminated polybutadiene, ureidopyrimidone, and aliphatic disulphide, showing remarkable mechanical and self-healing properties [28,29]; however, their adhesion was not tested. Additionally, some self-healing PU adhesives that need the addition of water or ethanol for adequate performance have also been developed [7,30,31].

Although polyether and polyester polyols are more commonly used for synthesizing PUs, the less common PUs formulated with polycarbonate polyols show superior elastomeric characteristics, commendable mechanical properties, and excellent weathering resistance [32]. Various self-healing PUs synthesized with polycarbonates have been documented. Some self-healing PUs formulated with these polyols needed heating at 80 °C for 40 s [33] or at 120 °C for 1 to 7 h [34]. Additionally, self-healing PUs made with blends of polycarbonate and propylene glycol polyols needed heating at 37 °C for 6 h [35]. In our research group, one PU that could self-heal at 20 °C made with polycarbonate polyol due to dynamic non-covalent exchange polycarbonate soft segment interactions was reported. These interactions were enhanced by the presence of free carbonate groups, a low number of free and hydrogen-bonded urethane groups, and the high mobility of the soft segments [36]. In a subsequent study [37], different PUs were synthesized using polycarbonate polyols of different molecular weights (500 and 2000 Da). The PUs formulated with polycarbonates of molecular weights 500 and 1000 Da exhibited rapid self-healing at 20 °C but had poor mechanical properties. Conversely, the PU formulated with a polycarbonate polyol of a 2000 Da molecular weight displayed good mechanical properties but did not exhibit self-healing at 20 °C.

The above analysis of the existing literature on self-healing PU adhesives has shown a broad range of self-healing efficiencies and adhesive strengths (100 kPa to 5.8 MPa). All these self-healing PU adhesives were synthesized by using somewhat sophisticated methods or chemicals. On the other hand, to our knowledge, PU self-healing adhesives synthesized with polycarbonate polyols have not been developed yet.

Therefore, this study aims to develop self-healing PUs with fast self-healing at 20 °C, potential good adhesion, and acceptable mechanical properties. Furthermore, our aim is the use of a simple synthesis procedure of the PUs which does not require sophisticated methods or chemicals. In order to reach these objectives, several PUs made with blends of polycarbonates of molecular weights 1000 and 2000 Da were synthesized. Because the PU formulated with a 1000 Da polycarbonate shows fast self-healing at 20 °C but has poor mechanical properties, and the PU formulated with a 2000 Da polycarbonate has good mechanical properties but lacks self-healing, the PUs formulated with blends of both polycarbonate polyols may produce an adequate balance between self-healing and mechanical properties.

## 2. Materials and Methods

### 2.1. Materials

Several PUs were obtained with blends of 1000 and 2000 Da polycarbonates of 1,6-hexanediol (CD1000 and CD2000, respectively) (Covestro, Leverkusen, Germany). Additionally, 4,4’-methylene bis(cyclohexyl) isocyanate (HMDI) (90% purity, Sigma Aldrich Co., St. Louis, MO, USA) and a 1,4-butanediol (BD) chain extender (99% purity, Panreac Applichem^®^, Darmstadt, Germany) were used.

### 2.2. Preparation of PUs

The synthesis procedure of the PUs was quite simple and did not require the use of sophisticated methods or chemicals.

The PUs were produced with the one-shot method, and they differ with variations in the compositions of the 1,6-hexanediol blend polycarbonates. The polyol or polyol blends (3.9062 g) and 1,4-butanediol (0.035 g) were placed in a container at 80 °C and mixed in a SpeedMixer DAC 150.1 FVZ-K double centrifuge (FlackTek Inc., Landrum, SC, USA) at 2400 rpm for 1 min. After an additional heating period of 10 min at 80 °C, HMDI isocyanate (1.0938 g) was added and stirred again at 2400 rpm for 1 min. The curing process of the PUs involved sequential 30 min stages at 50 °C, 60 °C, and 70 °C, with subsequent heating at 80 °C for 6 h. Then, the PUs were annealed at 85 °C for 1 h. A minimum of three 5 g batches of each PU were prepared, and good reproducibility was obtained.

The hard segment (HS) content in the PUs vary between 22 wt.% (C1000) and 13 wt.% (C2000), and the PUs made with CD1000 + CD2000 blends have intermediate HS contents (Table 1). Table 1 also shows the nomenclature of the PUs.

### 2.3. Experimental Techniques

Several experimental techniques were used for characterizing the structural, thermal, viscoelastic, mechanical, self-healing, and adhesion properties of the PUs.

The structure and chemical composition of the PUs was assessed by attenuated total reflectance infrared (ATR-IR) spectroscopy. An Alpha spectrometer (Bruker Optik GmbH, Ettlingen, Germany) equipped with a germanium prism was employed. Three ATR-IR spectra were collected for each PU.

The crystallinity of the PUs was determined by wide-angle X-ray diffraction (XRD) in a Bruker D8-Advance instrument (Bruker, Ettlingen, Germany) equipped with a copper anode and parallel beam geometry. All X-ray diffractograms have been normalized to the highest peak by means of Evaluation 14.0.0.0.0 software (version 2017).

The degree of micro-phase separation and the structure of the PUs were assessed by differential scanning calorimetry (DSC). Two consecutive heating cycles between −80 °C and 250 °C (heating rate: 10 °C/min) were performed under a nitrogen atmosphere in a DSC Q100 instrument (TA Instruments, New Castle, DE, USA).

The structural and thermal properties of the PUs were obtained by thermal gravimetric analysis (TGA) by using a TGA Q500 instrument (TA Instruments, New Castle, DE, USA). Furthermore, 9–10 mg PU was heated to 600 °C (heating rate: 10 °C/min).

Dynamic thermal mechanical analysis (DMA) analyzed the rheological/viscoelastic properties of the PUs. A single cantilever mode and a frequency of 1 Hz were applied. Rectangular PU pieces were heated from −100 °C to 80 °C (heating rate: 5 °C/min) in a DMA Q800 instrument (TA Instruments, New Castle, DE, USA).

The mechanical properties of dumbbell-shaped PU specimens were assessed according to the ASTM D 638 standard [38] in a Zwick/Roell Z005 universal testing machine (San Cugat del Vallés, Spain).

**Figure 1:**
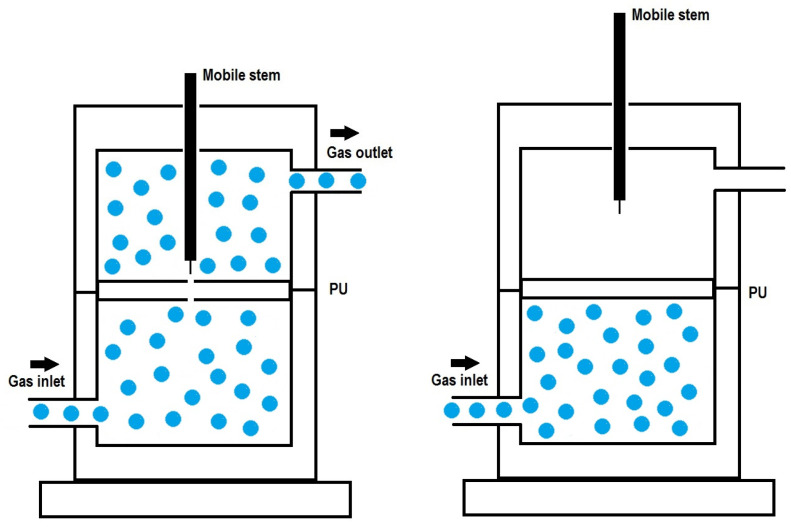
Scheme of the device employed to assess the self-healing ability and the kinetics of the self-healing of the PUs at 20 °C.

The self-healing at 20 °C was evaluated by placing PU disks inside a hermetic chamber which were fully perforated with a needle. Then, a continuous flow of nitrogen from the bottom to the top of the damaged PU piece was allowed, permitting the time and kinetics of self-healing to be determined [39]. Figure 1 illustrates the scheme of the device utilized for assessing the self-healing of the PUs.

Single lap-shear testing was performed on stainless steel/PU adhesive/stainless steel joints. Before joint formation, the surfaces of the stainless steel 304 pieces (30 mm × 150 mm × 1 mm) were mechanically abraded with a Scotch Brite^®^ green pad and were then wiped with isopropanol. The solvent was evaporated at room temperature for 5 min.

A droplet of melted polyurethane (0.28–0.36 g) was applied to a 20 mm × 30 mm side surface of one stainless steel specimen. Subsequently, the second stainless steel specimen was positioned on top, and a pressure of 2.5 MPa at 60 °C was applied for 1 min. The cure of the PU adhesive was made in a vacuum oven at 80 °C for 3 h. The adhesive thickness was 2.0–2.2 mm.

After a 72 h period, single lap-shear tests were conducted in a Zwick/Roell Z005 universal testing machine (San Cugat del Vallés, Spain) by using a pulling rate of 10 mm/min. Five replicates for each adhesive joint were carried out, and the results were averaged. The assessment of the loci of failure was made by visual inspection.

## 3. Results and Discussion

In our previous studies [37,40], it has been shown that the PUs formulated with 500 and 1000 Da polycarbonate polyols presented fast self-healing at 20 °C. It was concluded that the length of the soft segments and the interactions between them determined the self-healing of the PUs. Conversely, the PU obtained with a 2000 Da polycarbonate polyol did not show self-healing. Where the PUs formulated with 500 and 1000 Da polycarbonate polyols showed poor mechanical properties, the PU obtained with a 2000 Da polycarbonate polyol had high tensile strength and low elongation-at-break. Because the good mechanical properties of self-healing PUs are desirable, in this study, different PUs were synthesized with blends of 1000 and 2000 Da polycarbonate polyols to enhance the mechanical properties without sacrificing self-healing.

The adhesion properties of self-healing PUs have been poorly analyzed in the literature [5,7,8,24,27,30]. To our knowledge, the adhesion properties of self-healing PUs formulated with polycarbonate polyols have not been studied yet. Therefore, in this study, PUs showing fast self-healing at 20 °C, reasonable mechanical properties, and satisfactory adhesion properties were intended to be developed.

### 3.1. Structural Characterization and Degree of Micro-Phase Separation of PUs

The polycarbonates of 1,6-hexanediol with molecular weights of 1000 (CD1000) and 2000 Da (CD2000) have a linear structure differing in terms of the amount of carbonate groups (13 in CD1000 and 30 in CD2000) and the carbonate of 1,6-hexanediol units (6 in CD1000 and 13 in CD2000). Therefore, the soft segments in the PUs obtained with CD1000 + CD2000 mixtures will have a different amount of carbonate groups/carbonate of 1,6-hexanediol units depending on their polyols blend composition, i.e., the higher CD2000 content, the higher the amount of carbonate groups/carbonate of 1,6-hexanediol units and the higher the length of the soft segments. On the other hand, a decrease in the hard segment (HS) content is expected in the PUs obtained with CD1000 + CD2000 by increasing their CD2000 polyol content [41,42]. In fact, C1000 shows the highest HS content and C2000 the lowest, and the HS contents of the PUs obtained with CD1000 + CD2000 are intermediate (Table 1).

**Figure 2:**
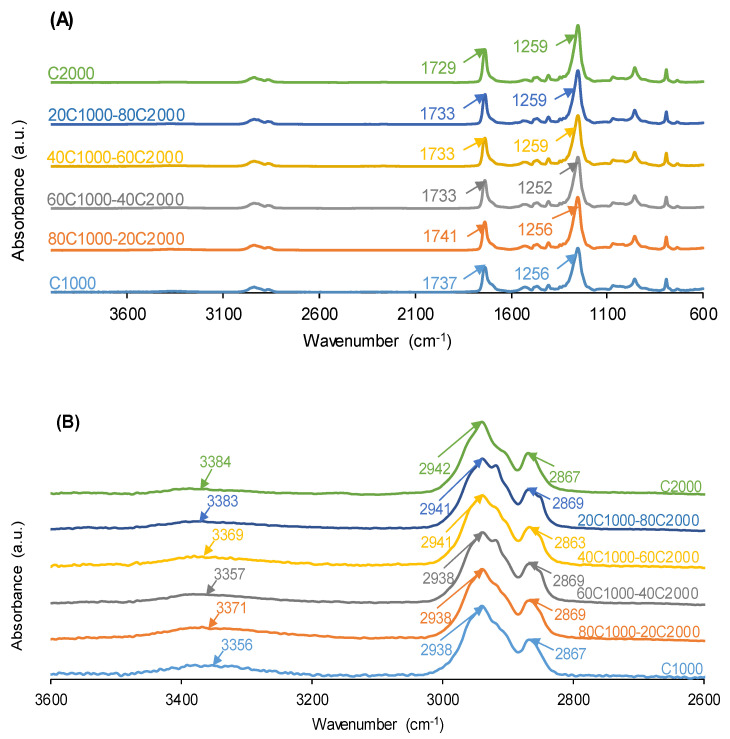
(**A**) ATR-IR spectra of PUs obtained with CD1000 + CD2000 mixtures. (**B**) 3600–2600 cm^−1^ region of the ATR-IR spectra.

**Figure 3:**
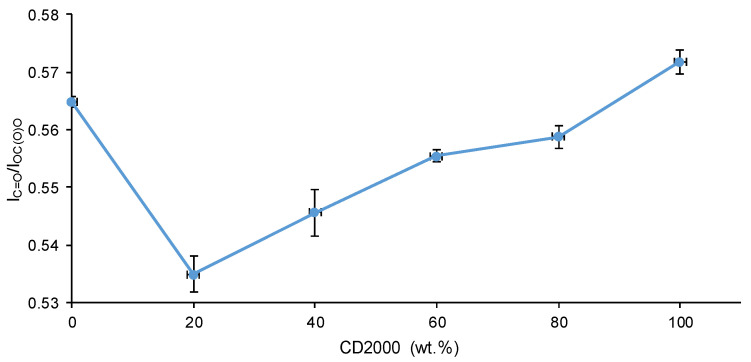
Variation of I_C=O_/I_OC(O)O_ ratios of PUs obtained with CD1000 + CD2000 mixtures with respect to their CD2000 polyol content.

The ATR-IR spectra of the PUs are influenced by their HS content and the number of carbonate species in the soft segments. All ATR-IR spectra show the same absorption bands: N-H stretching at 3356–3384 cm^−1^, C=O stretching of urethane and urea groups at 1729–1737 cm^−1^, C-H stretching at 2938–2942 and 2863–2869 cm^−1^, and O-C(O)-O stretching of carbonate groups at 1256–1259 cm^−1^ (Figure 2). The wavenumbers of the C=O bands are higher in 80C1000-20C2000 (1741 cm^−1^) and C1000 (1737 cm^−1^), lower in C2000 (1729 cm^−1^), and gradually decrease by increasing the CD2000 polyol content in the PUs. This trend agrees well with that of the HS contents of the PUs. Furthermore, the ratio of the intensities of the C=O and the O-C(O)-O stretching bands—I_C=O_/I_OC(O)O_—in the ATR-IR spectra rises as the CD2000 content in the PUs obtained with CD1000 + CD2000 mixtures increases, due to the greater quantity of carbonate species (Figure 3). However, the I_C=O_/I_OC(O)O_ ratio is higher in C1000 than in 80C1000-20C2000, indicating a structural change caused by the intercalation of 20 wt.% CD2000 soft segments among the dominant CD1000 soft segments in 80C1000-20C2000.

The amounts of carbonyl species (urethane, urea, and carbonate) in the PUs would change as a function of their relative percentages of CD1000 and CD2000 polyols. The calculation of C=O species in the PUs were determined through the curve fitting of the carbonyl region (1600–1800 cm⁻^1^) of the ATR-IR spectra. The individual carbonyl peaks were fitted to a Gaussian function, which was free from intensity and full width at half-maximum. The wavelengths of the different individual C=O species in the PUs were selected according to prior studies in the literature [36,43]. Thus, free C=O in polycarbonates was fitted at 1740 cm^−1^, free urethane at 1730 cm^−1^, carbonyl–carbonyl interactions between soft segments and hydrogen-bonded urethane groups at 1712 cm^−1^, free urea at 1699 cm^−1^, and hydrogen-bonded urea at 1660 cm^−1^. Even though no amines were employed in the synthesis of the PUs, the cure process took place in air and urea groups were formed.

**Figure 4:**
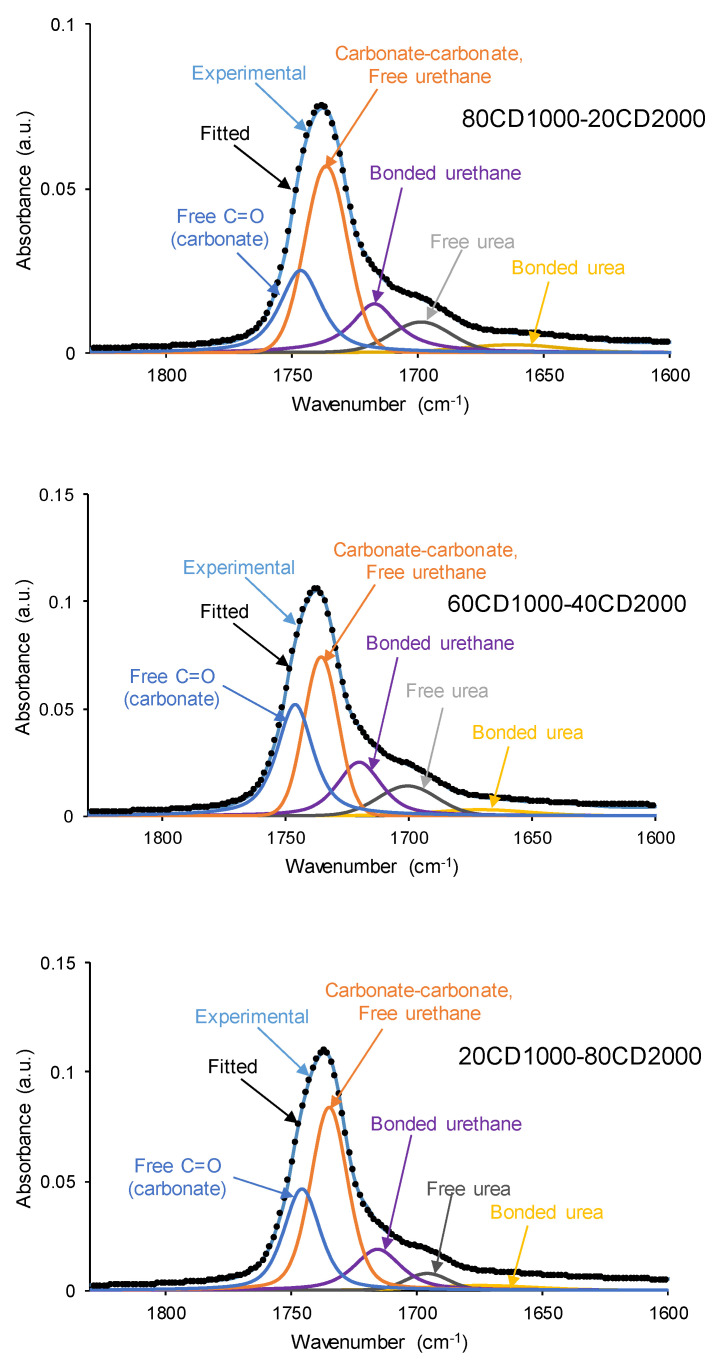
Curve fitting of the carbonyl stretching region of the ATR-IR spectra of some PUs obtained with CD1000 + CD2000 mixtures.

Figure 4 and Figure S1 of the Supplementary Materials file show the curve fitting of the carbonyl region of the ATR-IR spectra of the PUs. All PUs exhibit five different C=O species: free C=O of carbonate (1743–1745 cm^−1^), free urethane and carbonate–carbonate interactions (1733–1735 cm^−1^), hydrogen-bonded urethane (1713–1725 cm^−1^), free urea (1695–1697 cm^−1^), and hydrogen-bonded urea (1654–1664 cm^−1^). According to Table 2, the lowest percentages of free C=O species correspond to C1000 and 80C1000-20C2000, i.e., they have significant dipole carbonate–carbonate interactions. An increase in the number of free C=O species in the PUs as the CD2000 soft segments content increases can be anticipated, and it is shown in Table 2. However, 60C1000-40C2000 is an exception because it exhibits a higher percentage of free C=O species (33%) than expected, and this is an indication of the existence of a particular structure. Furthermore, free urethane and carbonate–carbonate species are dominant in all PUs, and their amounts vary between 34 and 48%.

The existence of C-H···O intermolecular interactions between carbonate groups has been demonstrated elsewhere [44]. The increase in the CD2000 soft segments in the PUs should decrease the amount of free urethane species, but, at the same time, the amount of carbonate–carbonate species should increase. However, the percentage of free urethane and carbonate–carbonate species is higher than expected in 80C1000-20C2000 because the intercalation of only 20% CD2000 soft segments favors the interactions between carbonate groups. Similarly, the amount of free urethane and carbonate–carbonate species in 20C1000-80C2000 is higher than in C2000 because the addition of only 20% CD1000 soft segments favors the carbonate–carbonate interactions. In fact, the interactions between similar carbonates are less stable than those among the carbonates of different natures [44]. Furthermore, the lowest percentages of free urethane and carbonate–carbonate species correspond to 60C2000-40C1000, which also has a high percentage of free C=O species, indicating a particular structure.

The percentage of hydrogen-bonded urethane groups is somewhat similar (14–18%) in all PUs, except in C2000, in which it is lower (Table 2). The amount of free urea is higher in C1000 and lower in 20C1000-80C2000, and the rest of PUs show similar percentages (10–12%). The amount of hydrogen-bonded urea is low and similar (3–4%) in all PUs, except in C1000, in which it is higher (9%).

**Figure 5:**
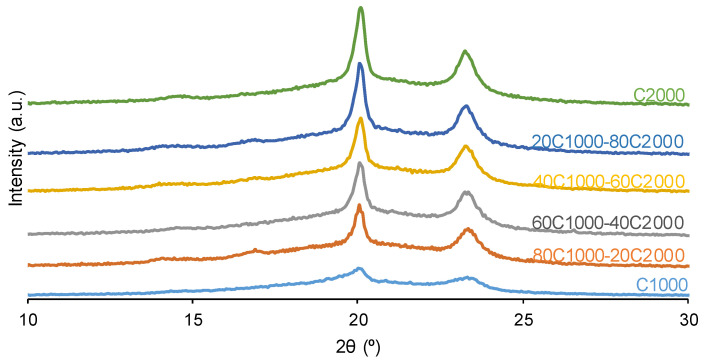
X-ray diffractograms of PUs obtained with CD1000 + CD2000 mixtures.

**Figure 6:**
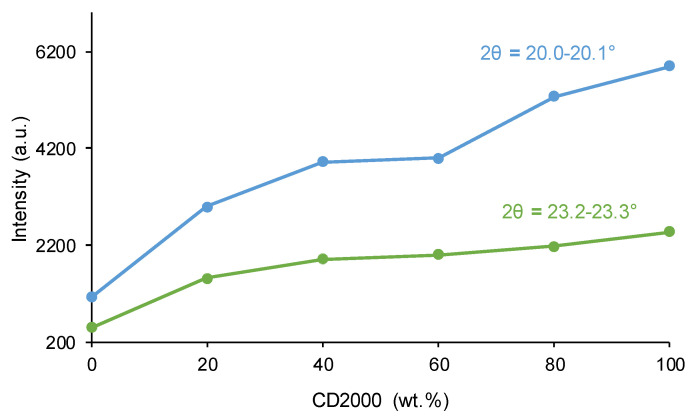
Variation in the intensities of the crystalline peaks at different 2θ values of PUs obtained with CD1000 + CD2000 mixtures as a function of their CD2000 polyol content. X-ray diffractograms.

The interactions among polycarbonate soft segments impart crystallinity to the PUs [36]. The X-ray diffractograms (Figure 5) of the PUs were adjusted to the highest peak by using Evaluation 14.0.0.0 software. All X-ray diffractograms displayed in Figure 5 exhibit wide halos (amorphous contributions), over which crystalline peaks can be distinguished. These two contributions in the X-ray diffractogram of 20CD1000-80CD2000, taken as typical example, can be distinguished in Figure S2 of the Supplementary Materials file. The intensity values of Figure 6 correspond only to the crystalline peaks, leaving out the amorphous contribution. Figure 5 shows two diffraction peaks at 2θ values of 20.0–20.1° and 23.2–23.3°, whose intensities increase as the content of the CD2000 soft segments rises (Figure 6). Furthermore, 80C1000-20C2000 shows a noticeable crystallinity with respect to C1000 (Figure 6) because the intercalation of 20% CD2000 soft segments favors the interactions between CD1000 soft segments. This agrees well with the high percentage of carbonate–carbonate interactions at 1733–1735 cm^−1^ in the ATR-IR spectrum of 80C1000-20C2000 (Table 2). On the other hand, the increase in the crystallinity in the other PUs is continuous and gradual as their CD2000 soft segment content increases. However, the intensity of the diffraction peak at 2θ = 20.1° of 60C1000-40C2000 is somewhat lower because of its high percentage of free C=O species and its low percentage of carbonate–carbonate species, in agreement with its ATR-IR spectrum (Table 2).

**Figure 7:**
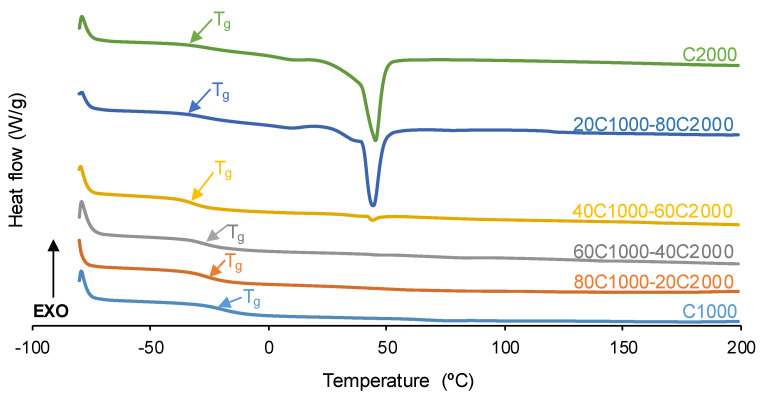
DSC curves of PUs obtained with CD1000 + CD2000 mixtures. First heating run.

The structure of the PUs was also evaluated by DSC. The glass transition temperature (T_g_) of the soft segments is distinguished in Figure 7 and decreases by raising the CD2000 soft segments content in the PUs (Table 3). The heat capacity at a constant pressure (∆c_p_) in the glass transition of the PUs increases when they contain 20–60% CD2000 soft segments, due to the greater number of interactions among the polycarbonate soft segments; furthermore, the ∆c_p_ values are lower in 20C1000-80C2000 and C2000. The DSC curves of these PUs show the cold crystallization and melting of the soft segments (Table 3). Furthermore, 20C1000-80C2000 and C2000 exhibit high crystallinity (X-ray diffractograms—Figure 5). Therefore, the mobility of the polycarbonate soft segments in 20C1000-80C2000 and C2000 is more restricted than in the other PUs, and the self-healing should not be favored.

Following a cool down to −80 °C, a second DSC heating run was conducted. The DSC curves (Figure S3 of the Supplementary Materials file) show the glass transitions of the soft (T_ss_) and hard (T_hs_) segments. In general, the T_ss_ values decrease and the T_ss_ values increase by increasing the CD2000 content in the PUs (Table 4). Thus, C2000 shows the lowest T_ss_ value and the highest T_hs_ value, so this PU exhibits the highest micro-phase separation. In general, the micro-phase separation in the PUs increases by increasing their CD2000 soft segment content.

**Figure 8:**
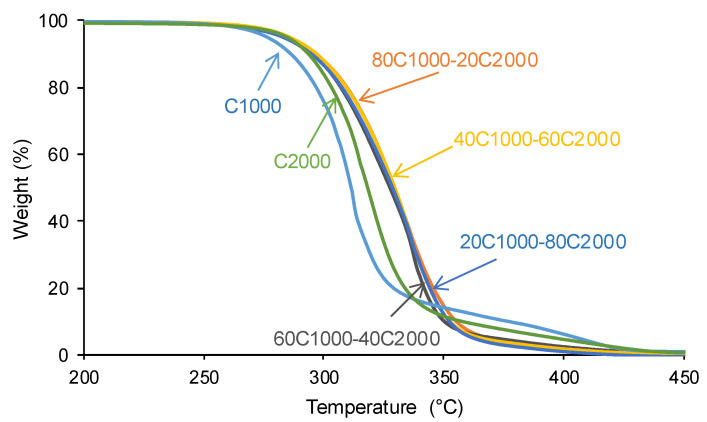
TGA curves of PUs obtained with CD1000 + CD2000 mixtures.

The interactions between the CD1000 and CD2000 soft segments would affect the TGA curves of the PUs. Figure 8 shows that the TGA curves of C1000 and C2000 appear at lower temperatures than those of the PUs obtained with CD1000 + CD2000 blends. The TGA curve of C2000 appears at a higher temperature than the one of C1000 due to the longer CD2000 soft segments. All PUs obtained with CD1000 + CD2000 blends exhibit very similar TGA curves, and, therefore, they have similar temperatures of maximum thermal decomposition (334–337 °C) and temperatures at which 50% of the mass is lost (324–330 °C) (Table S1 of the Supplementary Materials file).

**Figure 9:**
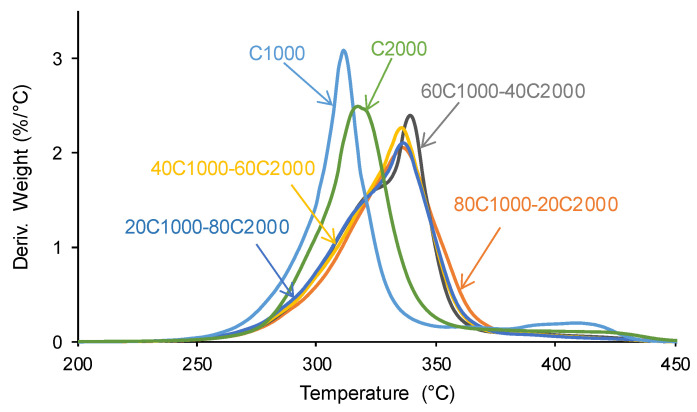
Derivatives of the TGA curves of PUs obtained with CD1000 + CD2000 mixtures. TGA experiments.

The thermal decompositions in the PUs are more clearly evidenced in the derivatives of the TGA curves. Figure 9 and Table S2 of the Supplementary Materials file show two thermal degradations of the soft and hard segments in C1000 (311 °C and 409 °C) and C2000 (317 °C and 421 °C); the thermal decomposition temperatures are higher in C2000 because of its higher number of carbonate groups. All PUs formulated with CD1000 + CD2000 blends show one additional thermal decomposition at 334–337 °C with weight losses of 41–50 wt.%, which is associated with the interactions among the carbonate groups of the CD1000 and CD2000 soft segments (mix phase).

**Figure 10:**
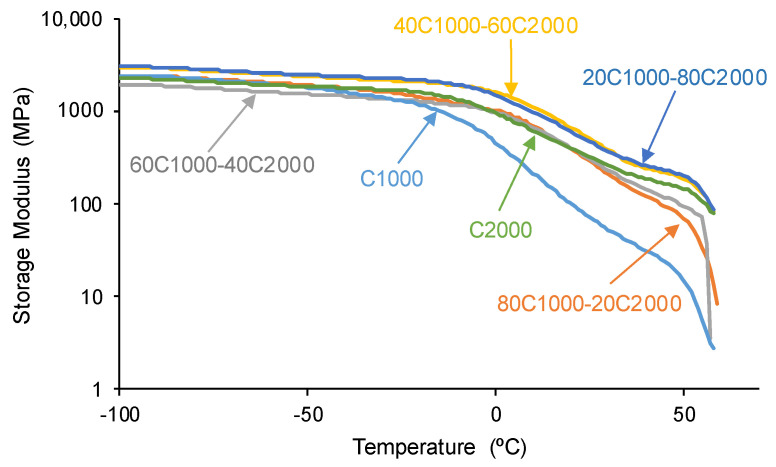
Variation in the storage (E′) moduli as a function of the temperature for PUs obtained with CD1000 + CD2000 mixtures. DMA experiments.

**Figure 11:**
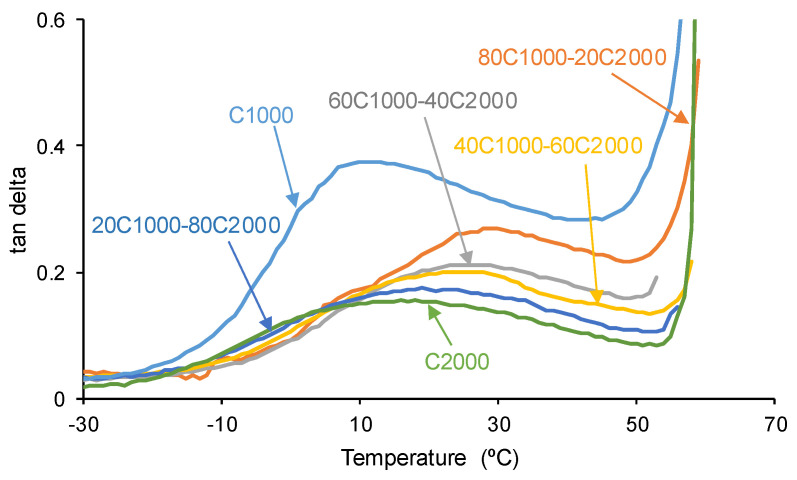
Variation in tan delta as a function of temperature for PUs obtained with CD1000 + CD2000 mixtures. DMA experiments.

The viscoelastic properties of the PUs are also affected by the interactions among polycarbonate soft segments of varying lengths [45]. According to Figure 10, upon increasing the temperature, the most important decrease in the storage moduli corresponds to C1000, and the storage moduli of the PUs made with CD1000 + CD2000 blends are higher, particularly in the region of the rubbery plateau. The highest storage moduli values correspond to the PUs obtained with 20–40% CD1000 + 80–20% CD2000. On the other hand, the tan delta vs. temperature plots of the PUs (Figure 11) reveal one structural relaxation, in which the tan delta values and temperatures in the maximum tan delta vary depending on the polyols mixture compositions. As the content of the CD2000 soft segments in the PUs increases, the tan delta values decrease steadily from 0.38 to 0.16 (Table 5), indicating enhanced interactions among the polycarbonate soft segments and increased crystallinity. In other words, whereas C1000 and 80C1000-20C2000 exhibit a dominant rheological viscous regime, the rest of the PUs exhibit a dominant rheological elastic regime. Furthermore, the temperatures of the maximum tan delta (T_tan delta_) in C1000 and C2000 are lower (10–17 °C) than in the PUs obtained with CD1000 + CD2000 blends (24–30 °C) due to more net interactions between the polycarbonate soft segments of a different nature than the ones of a similar nature [44] and the mixed phase of polycarbonate soft segments of different molecular weights.

### 3.2. Mechanical Properties of the PUs

**Figure 12:**
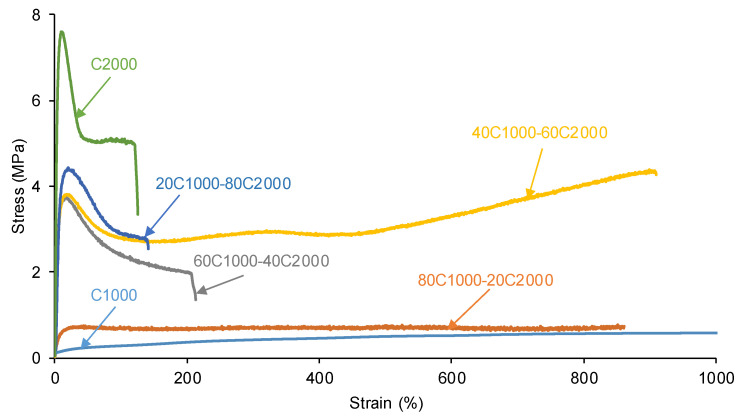
Stress–strain curves of PUs obtained with CD1000 + CD2000 mixtures.

The mechanical properties of the PUs were determined by stress–strain tests. According to Figure 12, the mechanical properties of the PUs increase by increasing their CD2000 soft segment content. The stress–strain curves of C1000 and 80C1000-20C2000 correspond to elastomeric materials (low moduli and high elongation-at-break) in agreement with their dominant rheological viscous regime. On the other hand, the rest of the PUs have a more pronounced yield point as the content of the CD2000 soft segments increases. Similarly, the Young modulus and tensile strength values of the PUs increase by increasing their CD2000 soft segment content (Table S3 of Supplementary Materials file). Interestingly, the elongation-at-break of 40C1000-60C2000 is higher (892%) than expected.

### 3.3. Self-Healing Assessment of the PUs at 20 °C

The development of self-healing PUs at room temperature by simple and inexpensive methods is very attractive for industrial manufacturing [46].

The self-healing of PUs at 20 °C obtained with polycarbonate polyols is favored by the availability of free carbonate groups, carbonate–carbonate interactions, small micro-phase separation and crystallinity, as well as higher mobility of the soft segments [36].

**Figure 13:**
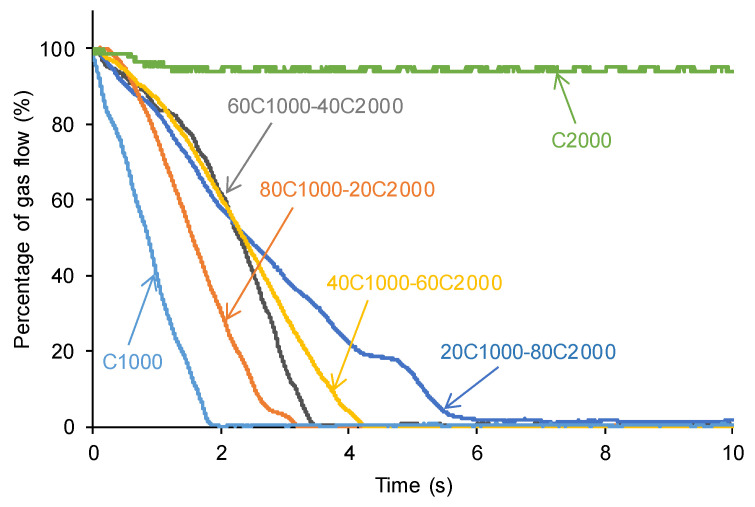
Self-healing kinetics at 20 °C of PUs obtained with CD1000 + CD2000 mixtures.

**Figure 14:**
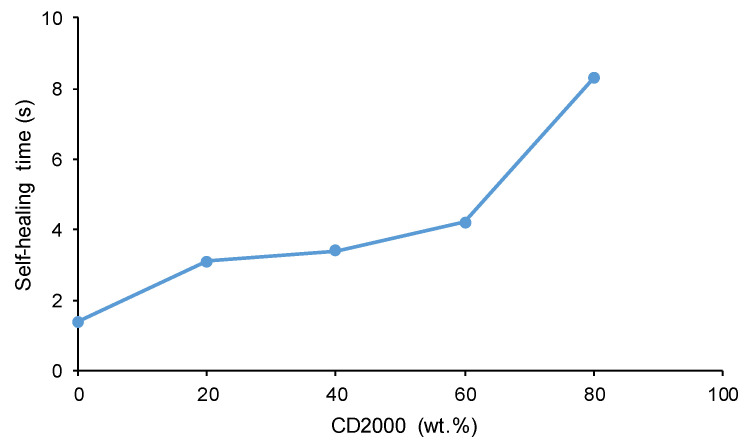
Variation in the self-healing time at 20 °C of PUs obtained with CD1000+CD2000 mixtures as a function of their CD2000 polyol content.

Figure 13 illustrates the self-healing kinetics of PUs obtained with CD1000 + CD2000 mixtures. The faster self-healing kinetics and shorter healing times are observed in C1000 (1.4 s), while CD2000 shows no self-healing at 20 °C. The increase in the content of CD2000 soft segments slows down the self-healing kinetics and extends the self-healing time of the PUs more noticeably in 20C1000-80C2000 (8.3 s) (Figure 14). Therefore, the presence of CD1000 soft segments, even in a small amount, imparts self-healing to the PUs, and the higher the amount of CD1000 soft segments, the faster the kinetics and the shorter the self-healing time. This trend agrees well with the experimental evidences of ATR-IR, X-ray diffraction, and DSC. Thus, C1000 and 80C1000-20C2000 exhibit fast kinetics and short self-healing times (1.4–3.1 s) because of the existence of free carbonate groups and important carbonate–carbonate interactions; furthermore, these PUs have low micro-phase separation and a dominant rheological viscous regime. On the contrary, 20C1000-80C2000 exhibits an important amount of free carbonate groups and interactions between carbonate groups, but also shows high micro-phase separation and high crystallinity; therefore, the kinetics of self-healing are slower and the self-healing time is longer. Interestingly, although 20C1000-80C2000 exhibits cool crystallization and low tan delta values, self-healing at 20 °C occurs.

The PUs obtained with CD1000 + CD2000 blends were synthesized by using a very simple and low-cost procedure, i.e., only four easily available precursors were used, and mild reaction conditions were set. In comparison, other self-healing PUs require laser excitation [47] or sophisticated reactants—5-methyl-5-benzyloxycarbonyl-1, 3-dioxan-2-one [35]; 1,8-menthane diamine (MD) and bis(2-hydroxyethyl)disulfide [48]; and bio-based epoxy resin and poly(dimethyl siloxane)-based vitrimer [49]. Conversely, the PUs obtained with CD1000 + CD2000 demonstrate rapid self-healing at room temperature. Although the self-healing evaluation method of these PUs differs from those used for other self-healing PUs, they show considerably superior performances. For instance, Dong et al. developed PU elastomers that self-healed after 30 min at 40 °C [48], and the PUs synthetized by Wang et al. self-healed after 2 h at 120 °C [47]. Zhang et al. synthesized PUs with aliphatic polycarbonates that self-healed after 6 h at 37 °C [35], and the PUs synthesized by Samyn et al. exhibited self-healing after 24 h at 50 °C [49].

On the other hand, it remains a challenge to obtain PUs with high mechanical properties and fast self-healing at room temperature. In fact, the PUs formulated with CD1000 + CD2000 blends exhibit lower mechanical properties (tensile strength values = 0.5–4.3 MPa) than the ones of other self-healing PUs (24.8–38 MPa) [47,48], but those have faster self-healing.

### 3.4. Proposed Mechanism of Self-Healing at 20 °C in PUs Obtained with CD1000 + CD2000 Blends

**Figure 15:**
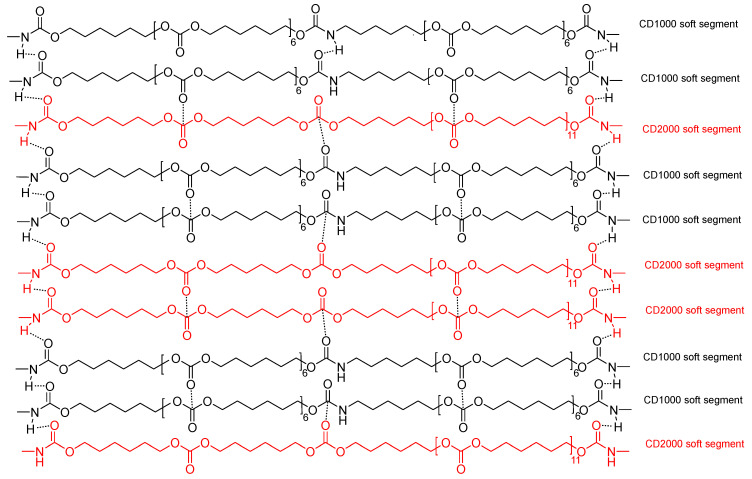
Scheme of the interactions among polycarbonate soft segments of different molecular weights in 60C1000-40C2000. CD1000 soft segments are shown in black and CD2000 soft segments are shown in red.

Based on the previous experimental results, a dynamic non-covalent exchange self-healing mechanism among polycarbonate soft segments of different molecular weights can be proposed. Taking as a typical example the structure of 60C1000-40C2000, Figure 15 shows how the intercalation of CD1000 soft segments favors the interactions between urethane/urea and carbonate groups by hydrogen bonds with CD2000 soft segments, causing the disruption of the carbonate–carbonate interactions. Simultaneously, the number of hydrogen-bonded urethane groups between CD1000 and CD2000 soft segments is reduced. Consequently, the greater number of carbonate–carbonate interactions and hydrogen-bonded urethane groups in C1000 accounts for its quicker kinetics and shorter self-healing time as compared to the other PUs. Furthermore, the intercalation of CD2000 soft segments decreases the number of carbonate–carbonate interactions, hydrogen-bonded urethane groups, and carbonates of the CD1000 soft segments, which justifies slower kinetics and shorter self-healing time. Thus, upon the application of stress or damage, the weak hydrogen bonds and carbonate–carbonate interactions are broken. Upon removing the stress, the mobile C1000 soft segments reform the broken interactions and self-healing can occur.

### 3.5. Adhesion of Stainless Steel/PU Adhesive/Stainless Steel Joints

In previous recent studies [5,7,8,24,27,30], self-healing PU adhesives have been synthesized, and their adhesion properties have been tested by single lap-shear tests. A broad range of shear values between 51.6 kPa and 5.69 MPa has been recorded.

**Figure 16:**
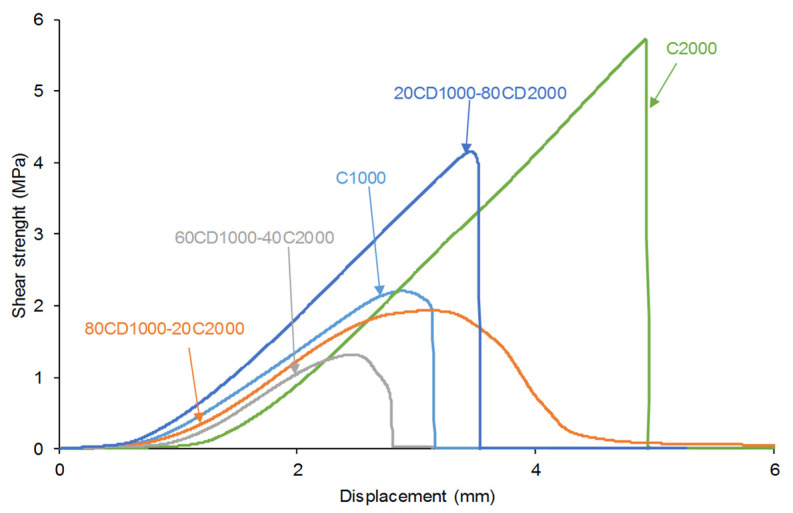
Shear strength vs. displacement curves of stainless steel/PU adhesive/stainless steel joints.

The adhesion of the PU adhesives made with CD1000 + CD2000 blends was determined by single lap-shear tests of stainless steel/PU adhesive/stainless steel joints. The shapes of the curves of the shear strength vs. the displacement of the joints made with 20C1000-80C2000 and C2000 are typical of structural adhesive joints [50], i.e., the shear strength increases by increasing the displacement until a maximum shear strength is reached, and then, the joint is de-bonded and the shear strength suddenly decreases to zero (Figure 16). However, in the curves of the joints made with 80C1000-20C2000 and 60C1000-40C2000, after reaching the maximum shear strength, the shear strength declined gradually (Figure 16). This may be an indication of the existence of self-healing in these joints because once the adhesive is detached from the substrate during the lap-shear test, it seems to re-join again, losing gradually shear strength.

**Figure 17:**
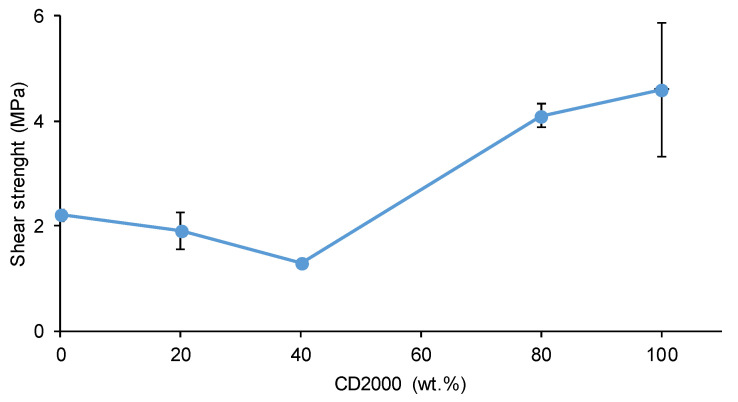
Variation in the shear strength of stainless steel/PU adhesive/stainless steel joints as a function of the CD2000 polyol content in the PU.

The shear strength values of the joints made with PU adhesives synthesized with CD1000 + CD2000 mixtures range between 1.3 and 4.6 MPa (Figure 17), values which are quite acceptable for polyurethanes. In fact, these shear strength values are comparable with the shear strength values (5.69 MPa) reported by Wang et al. [5] and Longfang et al. (1.73 MPa) [7]. Furthermore, the shear strength values of the joints made with PU adhesives synthesized with CD1000 + CD2000 mixtures are significantly higher than the ones obtained by Li et al. (0.9 MPa) [30] and Xu et al. (51.6 kPa) [27].

The joints made with C1000 and 80C1000-20C2000 adhesives show similar shear strength values (1.9–2.2 MPa), and they exhibit a mixed failure, mainly cohesive in the adhesive. The lowest shear strength (1.3 MPa) corresponds to the joint made with 60C1000-40C2000 adhesive in which a mixed failure, mainly adhesion failure, is obtained. The lowest adhesive strength of 60C1000-40C2000 agrees well with its particular structure derived from the competition regarding the interactions of the polycarbonate soft segment of different molecular weights. On the other hand, in the joints made with 20C1000-80C2000 and C2000 adhesives, the shear strengths increase noticeably (4.1–4.6 MPa), and 20C1000-80C2000 shows self-healing at 20 °C, high mechanical properties, and dominant elastic rheological behavior. The joints exhibit a mixed failure, mainly of adhesion in the joint made with the 20C1000-80C2000 adhesive and mainly cohesion in the adhesive in the joint made with the C2000 adhesive.

## 4. Conclusions

Polyurethanes showing self-healing at 20 °C, acceptable mechanical properties, and high adhesion were synthesized by using blends of polycarbonate polyols of different molecular weights (CD1000 + CD2000). The synthesis procedure of these PUs was quite simple and did not require the use of sophisticated methods or chemicals. The increase in CD2000 soft segments caused slower kinetics and longer self-healing times in the PUs, as well as higher mechanical and adhesion properties, due to a dominant rheological elastic behavior, higher crystallinity, and greater micro-phase separation.

The PUs obtained with CD1000 + CD2000 blends exhibited one additional thermal decomposition at 334–337 °C due to a new mix phase formed by interactions between the carbonate groups of CD1000 and CD2000 soft segments. Due to the existence of the mix phase, the storage moduli and the temperatures of the maximum tan delta of the PUs obtained with CD1000 + CD2000 blends were higher than the ones of C1000 and C2000.

The intercalation of C1000 soft segments favored the formation of hydrogen bonds between urethane/urea and the carbonate groups of C2000 soft segments, disrupting the interactions between carbonate groups. At the same time, the amount of hydrogen-bonded urethane groups was reduced between C1000 and C2000 soft segments. Therefore, the greater number of carbonate–carbonate interactions and hydrogen-bonded urethane groups in C1000 justified its faster kinetics and short self-healing time. C1000 and 80C1000-20C2000 had the lowest percentages of free C=O species and a significant number of carbonate–carbonate interactions. Furthermore, 80C1000-20C2000 showed noticeable crystallinity with respect to C1000 because the intercalation of 20% CD2000 soft segments favored the urethane/urea–carbonate and carbonate–carbonate interactions between the soft segments.

The Young modulus and tensile strength values of the PUs increased by increasing their CD2000 soft segment content. The shear strength values of the joints made with PU adhesives synthesized with CD1000 + CD2000 mixtures ranged between 1.3 and 4.6 MPa, and they increased by increasing the CD2000 soft segments content. These shear strength values were quite acceptable for PU adhesives, and they were within the range of those in the existing literature.

## Figures and Tables

**Table 1:** Hard segments (HS) contents, nomenclature, and composition of PUs synthesized with CD1000 + CD2000 mixtures.

PU	Polyols Composition *	HS (wt.%)
C1000	CD1000	22
80C1000-20C2000	80% CD1000 + 20% CD2000	21
60C1000-40C2000	60% CD1000 + 40% CD2000	19
40C1000-60C2000	40% CD1000 + 60% CD2000	17
20C1000-80C2000	20% CD1000 + 80% CD2000	15
C2000	CD2000	13

* CD1000: Polycarbonate of 1,6-hexanediol—M_w_: 1000 Da; CD2000: Polycarbonate of 1,6-hexanediol—M_w_: 2000 Da.

**Table 2:** Amounts of C=O species in the carbonyl stretching region of the ATR-IR spectra of PUs obtained with CD1000 + CD2000 mixtures.

Wavenumber (cm^−1^)	Percentage (%)						Assignment
	C1000	80C1000-20C2000	60C1000-40C2000	40C1000-60C2000	20C1000-80C2000	C2000	
1654–1664	9	4	4	3	3	3	Bonded urea
1695–1697	15	10	11	12	4	12	Free urea
1713–1725	14	18	18	15	16	11	Bonded urethane
1733–1735	38	43	34	40	48	40	Carbonate-carbonate, free urethane
1743–1745	24	24	33	29	29	34	Free C=O (carbonate)

**Table 3:** Thermal events derived from the DSC curves of PUs obtained with CD1000 + CD2000 mixtures. First heating run.

PU	T_g_ (°C)	$∆c_{p}\;(J/g$ °C)	T_c_ (°C)	ΔH_c_ (J/g)	T_m_ (°C)	ΔH_m_ (J/g)
C1000	−21	0.29	-	-	-	-
80C1000-20C2000	−26	0.40	-	-	-	-
60C1000-40C2000	−27	0.35	-	-	-	-
40C1000-60C2000	−29	0.35	-	-	44	1
20C1000-80C2000	−29	0.22	21	2	44	14
C2000	−29	0.20	20	1	45	23

**Table 4:** Thermal events derived from the DSC curves of PUs obtained with CD1000 + CD2000 mixtures. Second heating run.

PU	T_ss_ (°C)	T_hs_ (°C)
C1000	−18	236
80C1000-20C2000	−23	231
60C1000-40C2000	−27	232
40C1000-60C2000	−31	235
20C1000-80C2000	−32	238
C2000	−36	240

**Table 5:** Tan delta values and temperatures at which tan delta reaches its maximum for PUs obtained with CD1000 + CD2000 mixtures. DMA experiments.

**PU**	**t** **an delta**	**T_tan delta_ (°C)**
C1000	0.38	10
80C1000-20C2000	0.27	30
60C1000-40C2000	0.21	26
40C1000-60C2000	0.20	26
20C1000-80C2000	0.17	24
C2000	0.16	17

## Data Availability

The original contributions presented in the study are included in the article/Supplementary Materials, further inquiries can be directed to the corresponding author.

## Supplementary Materials

The following supporting information can be downloaded at: https://www.mdpi.com/article/10.3390/ma17225532/s1. Figure S1. Curve fitting of the carbonyl stretching region of the ATR-IR spectra of some PUs; Figure S2. X-ray diffractogram of 20CD1000-80CD2000 showing the halo of the amorphous contribution and the crystalline peaks; Figure S3. DSC curves of PUs formulated with CD1000 + CD2000 mixtures. Second heating run; Table S1. Temperatures at which 50% (T_50%_) mass is lost and temperatures of maximum decomposition (T_max_) in PUs formulated with CD1000 + CD2000 mixtures. TGA experiments; Table S2. Temperatures and weight losses of the thermal degradations of PUs formulated with CD1000 + CD2000 mixtures. DTGA experiments; Table S3. Parameters obtained from the stress–strain curves of PUs formulated with CD1000 + CD2000 mixtures.
