# Supplementary material for: Polyurethanes Made with Blends of Polycarbonates with Different Molecular Weights Showing Adequate Mechanical and Adhesion Properties and Fast Self-Healing at Room Temperature
Source: Materials (Basel). 2024 Nov 13;17(22):5532. doi: 10.3390/ma17225532 (PMC11595875; doi:10.3390/ma17225532)
Supplement: Supplementary file 1 [file materials-17-05532-s001.zip › materials-3271428-supplementary.pdf]

materials

# Supplementary material
# Polyurethanes made with blends of polycarbonates with dif-ferent molecular weights showing fast self-healing at room temperature, and adequate mechanical and adhesion properties

Yuliet Paez-Amieva, Noemí Mateo-Oliveras, José Miguel Martín-Martínez

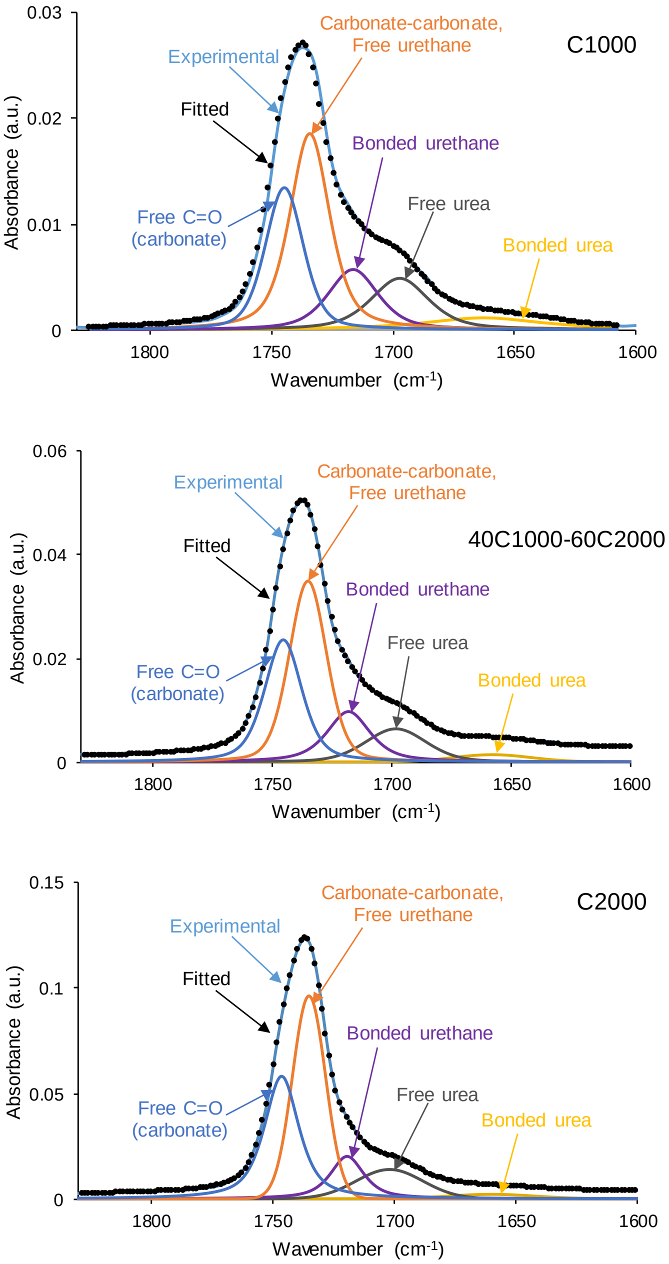


**Figure S1.** Curve fitting of the carbonyl stretching region of the ATR-IR spectra of some PUs.

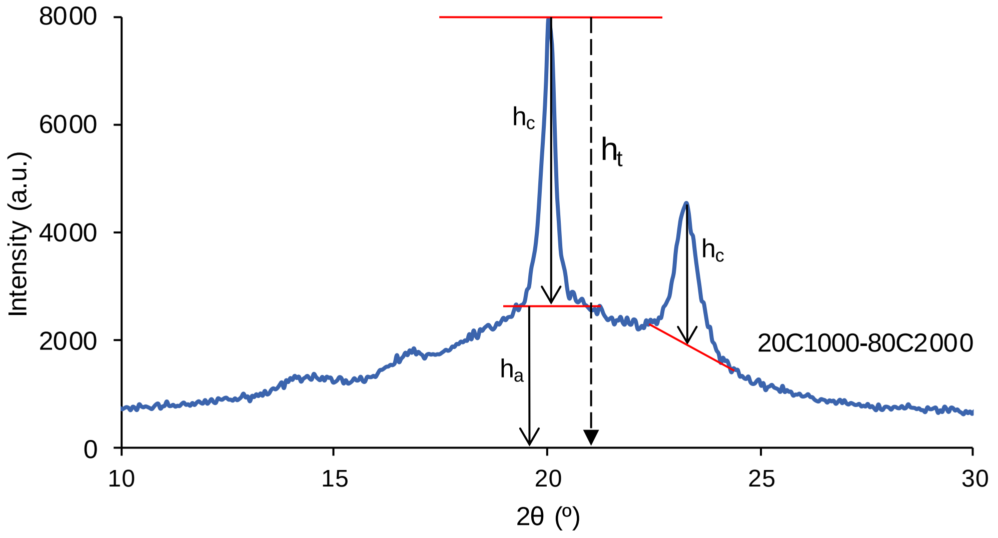


**Figure S2.** X-ray diffractogram of 20CD1000-80CD2000 showing the halo of the amorphous contribution and the crystalline peaks.

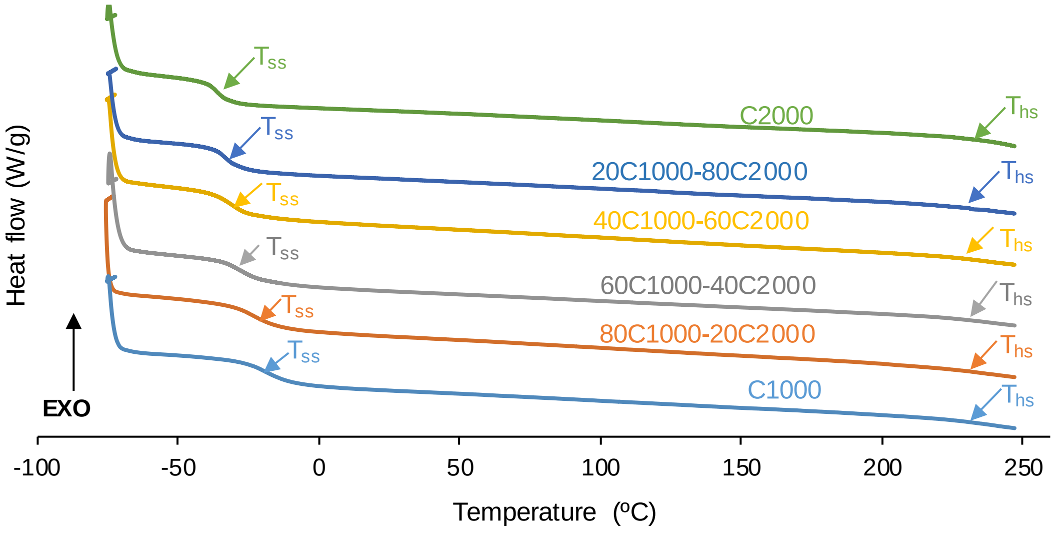


**Figure S3.** DSC curves of PUs made with CD1000+CD2000 mixtures. Second heating run.

**Table S1.** Temperatures at which 50 % ($T_{50\%}$) mass is lost and temperatures of maximum decomposition ($T_{max}$) in PUs made with CD1000+CD2000 mixtures. TGA experiments.

| PU | $T_{50\%}$ (°C) | $T_{max}$ (°C) |
|---|---|---|
| C1000 | 311 | 311 |
| 80C1000-20C2000 | 330 | 334 |
| 60C1000-40C2000 | 324 | 337 |
| 40C1000-60C2000 | 329 | 335 |
| 20C1000-80C2000 | 329 | 336 |
| C2000 | 318 | 317 |

**Table S2.** Temperatures and weight losses of the thermal degradations of PUs made with CD1000+CD2000 mixtures. DTGA experiments.

| PU | 1st degradation | | 2nd degradation | | 3rd degradation | | 4th degradation | |
|---|---|---|---|---|---|---|---|---|
| | $T_1$ (°C) | Weight $loss_1$ (%) | $T_2$ (°C) | Weight $loss_2$ (%) | $T_3$ (°C) | Weight $loss_3$ (%) | $T_4$ (°C) | Weight $loss_4$ (%) |
| C1000 | 292 | 30 | 311 | 60 | - | - | 412 | 10 |
| 80C1000-20C2000 | 282 | 9 | 323 | 42 | 335 | 45 | 398 | 4 |
| 60C1000-40C2000 | 282 | 11 | 318 | 42 | 335 | 43 | 410 | 4 |
| 40C1000-60C2000 | 289 | 14 | 320 | 33 | 332 | 50 | 392 | 3 |
| 20C1000-80C2000 | 291 | 9 | 316 | 46 | 337 | 42 | 409 | 3 |
| C2000 | 293 | 26 | 320 | 66 | - | - | 421 | 8 |

**Table S3.** Parameters obtained from the stress-strain curves of PUs made with CD1000+CD2000 mixtures.

| PU | Young modulus (kPa) | Yield point | | Break point | |
|---|---|---|---|---|---|
| | | $\sigma_y$ (kPa) | $\varepsilon_y$ (%) | $\sigma_b$ (kPa) | $\varepsilon_b$ (%) |
| C1000 | 60 | - | - | 590 | >1119 |
| 80C1000-20C2000 | 70 | - | - | 490 | > 870 |
| 60C1000-40C2000 | 730 | 3730 | 12 | 1960 | 201 |
| 40C1000-60C2000 | 820 | 3790 | 16 | 4340 | 892 |
| 20C1000-80C2000 | 790 | 4380 | 18 | 2740 | 138 |
| C2000 | 1800 | 8000 | 12 | 5000 | 119 |

**Disclaimer/Publisher's Note:** The statements, opinions and data contained in all publications are solely those of the individual author(s) and contributor(s) and not of MDPI and/or the editor(s). MDPI and/or the editor(s) disclaim responsibility for any injury to people or property resulting from any ideas, methods, instructions or products referred to in the content.
